# Tolerability and outcomes of neuroendocrine tumors treated with PRRT and SBRT

**DOI:** 10.1530/EO-24-0001

**Published:** 2024-06-27

**Authors:** Jose E Nunez, Sylvia Ng, Hanbo Chen, Simron Singh, Julie Hallet, Calvin Law, Sten Myrehaug

**Affiliations:** 1 Division of Medical Oncology, Odette Cancer Centre, Sunnybrook Health Sciences Centre, University of Toronto, Toronto, Ontario, Canada; 2 Department of Radiation Oncology, Odette Cancer Centre, Sunnybrook Health Sciences Centre, University of Toronto, Toronto, Ontario, Canada; 3 Department of Surgery, Odette Cancer Centre, Sunnybrook Health Sciences Centre, University of Toronto, Toronto, Ontario, Canada

**Keywords:** neuroendocrine, peptide receptor radionuclide therapy, stereotactic body radiation therapy

## Abstract

There is interest in optimizing peptide receptor radionuclide therapy (PRRT) for the management of metastatic neuroendocrine neoplasms (NEN). The addition of stereotactic body radiation therapy (SBRT) may provide synergistic benefits by targeting specific sites of disease that may represent areas of tumor heterogeneity. Little is known about the efficacy or potential toxicity of this approach; understanding the outcomes of patients treated with these two modalities in a sequential fashion will provide insights into the appropriateness of embarking on a combined therapy strategy. An institutional retrospective review of 21 patients with NEN treated with sequential PRRT and SBRT (64 targets) was performed. Median overall survival and progression-free survival were 19.6 months and 12.8 months, respectively. Median time to local recurrence at the SBRT site was not reached, with rates at 12 and 24 months of 1.8% and 5.9%, respectively. The toxicity profile remains favorable. Given the safety and efficacy of sequential SBRT and PRRT, further trials evaluating a concurrent treatment approach may be warranted.

## Introduction

Neuroendocrine neoplasms (NEN) constitute a heterogeneous cancer diagnosis, with treatment and patient outcomes remaining variable. Classification systems distinguish between well-differentiated NEN, which typically behave in a more indolent fashion, and poorly differentiated neuroendocrine carcinomas, which are generally more aggressive (Klimstra *et al.* 2019). Well-differentiated NEN may have a longer survival rate (Dasari *et al.* 2017, Chauhan *et al.* 2020), necessitating a multidisciplinary assessment of treatment sequencing in order to achieve a balance between tumor control, quality of life, and treatment toxicity.

Peptide receptor radionuclide therapy (PRRT) is a pillar in the treatment of progressive well-differentiated neuroendocrine tumors. ^177^Lu-DOTATATE is the only Food and Drug Administration-approved radioligand for the treatment of gastroenteropancreatic-neuroendocrine tumors. This approval is based on the significant improvement in progression-free survival (PFS) compared to high-dose long-acting octreotide, as demonstrated in the landmark NETTER-1 trial, and it is now the standard of care for progressive disease following somatostatin analog therapy (Strosberg *et al.* 2017). However, these tumors may have heterogeneous clonal subpopulations that contribute to instances of non-response, either through reduced cell-surface somatostatin receptors (SSTR) or other mechanisms of treatment resistance (Walter *et al.* 2018, Puranik *et al.* 2021). Furthermore, standard treatment entails a fixed activity of PPRT to be delivered; but the actual absorbed dose may vary across the entire metastatic burden of disease in a patient. Although there is interest in an individualized dosimetry approach to account for this variability, there is currently no standard approach to enhance PRRT delivery in this scenario.

Stereotactic body radiotherapy (SBRT) is the delivery of ultra-high dose per fraction radiation to small volumes with the intent of tumor ablation or to provide durable local control. This is a standard-of-care option for treating any solid tumor type when local control or symptomatic management is required (Schneider *et al.* 2018, Ohri *et al.* 2021, Sahgal *et al.* 2021). Additionally, it is being widely investigated as an option to boost gross oligometastatic disease in the context of standard-of-care systemic treatment (Palma *et al.* 2019, Wang *et al.* 2023). Specific to neuroendocrine tumors, SBRT is emerging as an effective treatment of both primary and metastatic disease (Chen *et al.* 2021, Hudson *et al.* 2022, Oliver *et al.* 2023) Furthermore, SBRT may assist in providing hormonal control in functional NEN (Myrehaug *et al.* 2020).

The combination of PRRT and SBRT in the treatment of neuroendocrine tumors may have a synergistic impact, improving overall efficacy. PRRT provides systemic radiation treatment targeting tumor cells utilizing SSTR2 receptors, whereas SBRT delivers ultra-high doses of radiation to a specific area irrespective of the underlying tumor biology. However, there is scarce data evaluating the potential synergies or toxicities of these therapies combined. There is interest in utilizing PRRT as the primary modality to treat widely metastatic disease, with the addition of SBRT to target areas of tumor heterogeneity, such as low SSTR2 expression or sites of more poorly differentiated disease (Singh *et al.* 2023). Additional research is required to confirm these potential benefits. Before developing and embarking on clinical trials, safety and efficacy of combined treatment with PRRT and SBRT need to be established. Given the lack of clinical trial protocols and published data on patients treated concurrently, insights into the appropriateness of this strategy may be taken from patients treated in a sequential fashion. This is the first series dedicated to evaluating patients treated with sequential PRRT and SBRT for locally advanced/metastatic NEN.

## Methods

### Patients

An institutional retrospective chart review was performed on patients with well-differentiated neuroendocrine neoplasms treated in the Susan Leslie Clinic for Neuroendocrine Tumors with both SBRT and PRRT between January 2013 and May 2023. Demographics, past treatment details, clinical/radiographic/laboratory data were abstracted from the patient’s clinical and radiation planning records. All techniques, data collection, and processing in this study adhere to the regulations of the Research Ethics Board of the Sunnybrook Research Institute. Treatment-related toxicity was evaluated using CTCAE v5.0. Since patients were not treated concurrently, toxicity was attributed to the treatment modality based on the timing of toxicity related to the treatment or known treatment risk. When applicable, RECIST v1.1 or SPINO criteria (Thibault *et al.* 2015) were utilized to evaluate treatment response.

### Peptide receptor radionuclide therapy

All patients receiving PRRT were evaluated in a multidisciplinary tumor board to ensure the appropriateness of therapy, following verification of SSTR+ disease on ^68^Ga-DOTATATE PET. Imaging with ^18^FDG was not utilized in this patient population. All patients were treated with ^177^Lu-DOTATATE for at least three cycles for consideration of evaluation. Patients were treated according to a standardized protocol, which included the infusion of a 2.5% lysine/2.5% arginine amino acid solution and antiemetic (Ondansetron 8 mg p.o.) prior to ^177^Lu-DOTATATE infusion, which is then administered over 30 min using a shielded infusion pump system. All patients were evaluated by the nuclear medicine radiation safety team to ensure adherence to institutional radiation safety protocols.

### Stereotactic body radiotherapy

SBRT was initiated before or following PRRT, either in the oligometastatic/oligoprogressive setting based on conventional cross-sectional imaging or for symptom palliation. All treatments were conducted in accordance with institutional guidelines for the delivery of ablative radiotherapy and underwent site-specific peer quality assurance. SBRT characteristics are delineated in Table 1.

**Table 1:** Stereotactic ablative body radiotherapy details.

Total targets treated	64
Primary	4
Liver	27
Spine	21
Non-spine bone	12
Median dose prescribed BED_10_ (range)	
Primary	53.8 (37.5–60)
Liver	100 (37.5–180)
Spine	67.2 (37.5–67.2)
Non-spine bone	56.2 (37.5–67.2)
Median BED_2_ spinal cord (range)	89.2 (37.5–112.4)
Median uninvolved liver volume (range)	1073.5 cm^3^ (538–2261.9)

BED, biologically effective dose.

### Statistical analysis

Statistical analyses were conducted using the R statistical programming language. For continuous variables with a normal distribution, the mean and s.d. were reported; for those without a normal distribution, the median and range were provided. We utilized the Kaplan–Meier models to estimate survival, and the log-rank test to compare survival rates between groups. A *P*-value of 0.05 or less was considered statistically significant. The start date of follow-up is the date of completion of the last of any recorded treatment (either SBRT or PRRT) to avoid immortal time bias.

## Results

### Outcomes

**Figure 1:**
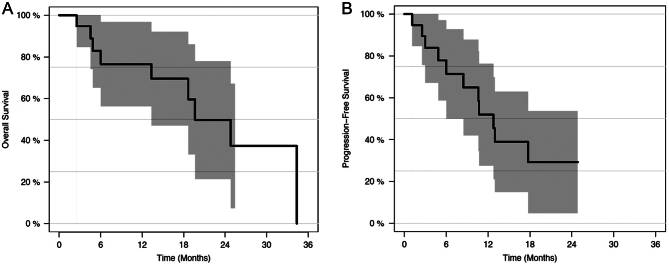
(A) Overall survival. (B) Progression-free survival.

Twenty-one patients with metastatic well-differentiated NEN treated with both PRRT and SBRT were identified, with a total of 64 individual targets treated with SBRT. Patient characteristics are shown in Table 2. SBRT and PRRT were delivered sequentially; SBRT was delivered prior to PRRT in 14 patients, after PRRT in three patients, and before/after PRRT in four patients. The median follow-up was 40 months (range 11–112 months). The median time between SBRT and PRRT was 9.2 months (range 0.9–58.5), while the median time between PRRT and SBRT was 20.8 months (range 9.5–45). Figure 1 illustrates overall survival (OS) and progression-free survival (PFS) for the entire patient cohort. Median OS in the overall population was 19.6 (95% CI: 13.3 – not reached) months. Overall median PFS was 12.8 (6.0 – not reached) months.

**Table 2:** Patient and treatment characteristics.

Variable	*n* = 21 patients
Median age at diagnosis (range) (years)	55 (40–77)
Median follow-up (range) (months)	40 (11–112)
Sex	
Female	7
Male	14
Primary	
Small intestine	7
Rectum	6
Pancreas	5
Other/Unknown	3
Grade	
1	5
2	14
3	2
Prior therapy	
Surgery – primary	9
Surgery – liver	4
Somatostatin analog	21
Liver-directed therapy	4
Targeted therapy	8
Conventional/palliative radiotherapy	3
Timing of SBRT related to PRRT	
Before	14
Following	3
Before and following	4
Total cycles of PRRT delivered	
3	2
4	19

PRRT, peptide receptor radionuclide therapy; SBRT, stereotactic body radiation therapy.

**Figure 2:**
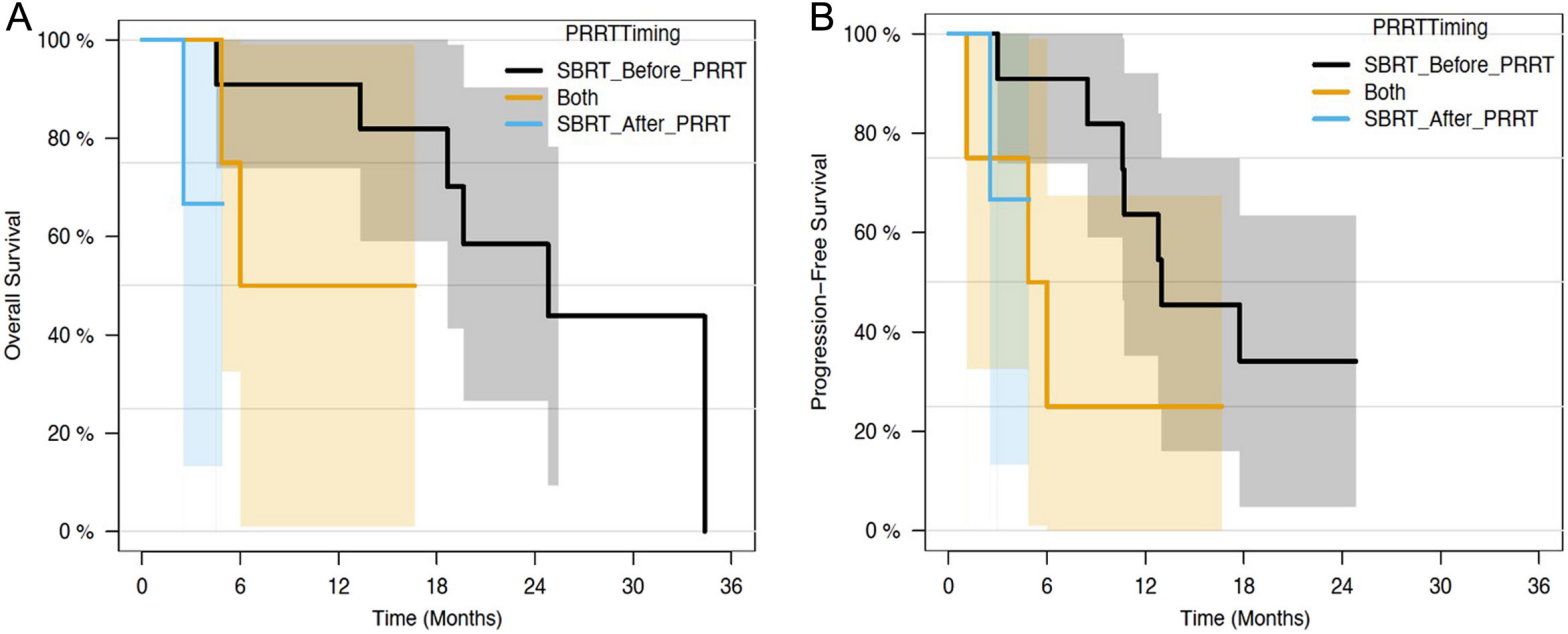
(A) Overall survival stratified by SBRT/PRRT timing. (B) Progression-free survival sratified by SBRT/PRRT timing.

Figure 2 demonstrates OS and PFS specific to timing of SBRT and PRRT. Those patients treated with SBRT prior to PRRT trend toward improved OS and PFS, which were not statistically significant on univariate analysis (data not shown).

**Figure 3:**
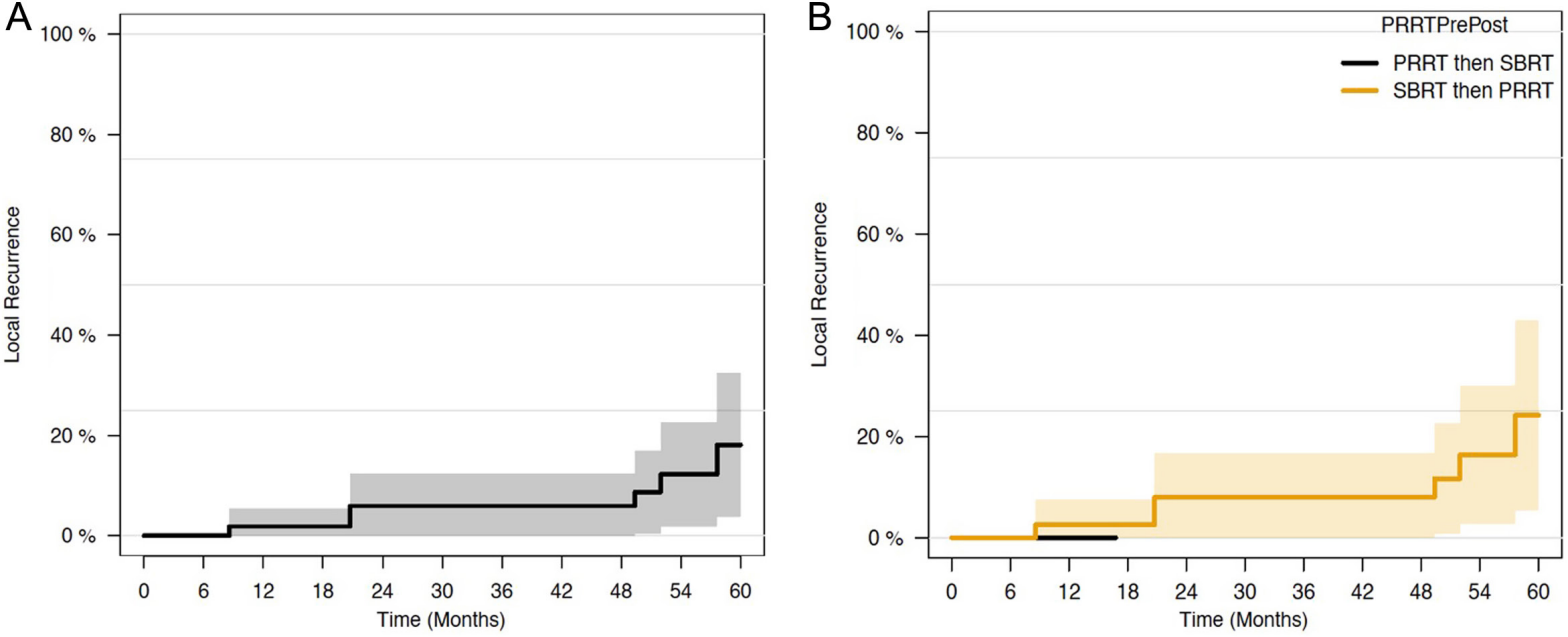
(A) Local recurrence rates in overall population. (B) Local recurrence rates stratified by SBRT/PRRT timing.

Median time to local recurrence was not reached, with rates at 12 and 24 months of 1.8% (0–5.4%) and 5.9% (0–12.4%), respectively. When evaluating the timing of SBRT relative to PRRT, no failures were seen in the cohort of patients treated with PRRT first and then subsequently with SBRT upon radiographic or clinical progression (Fig. 3). For those who had local recurrence, this occurred at a median of 22.8 months.

**Figure 4:**
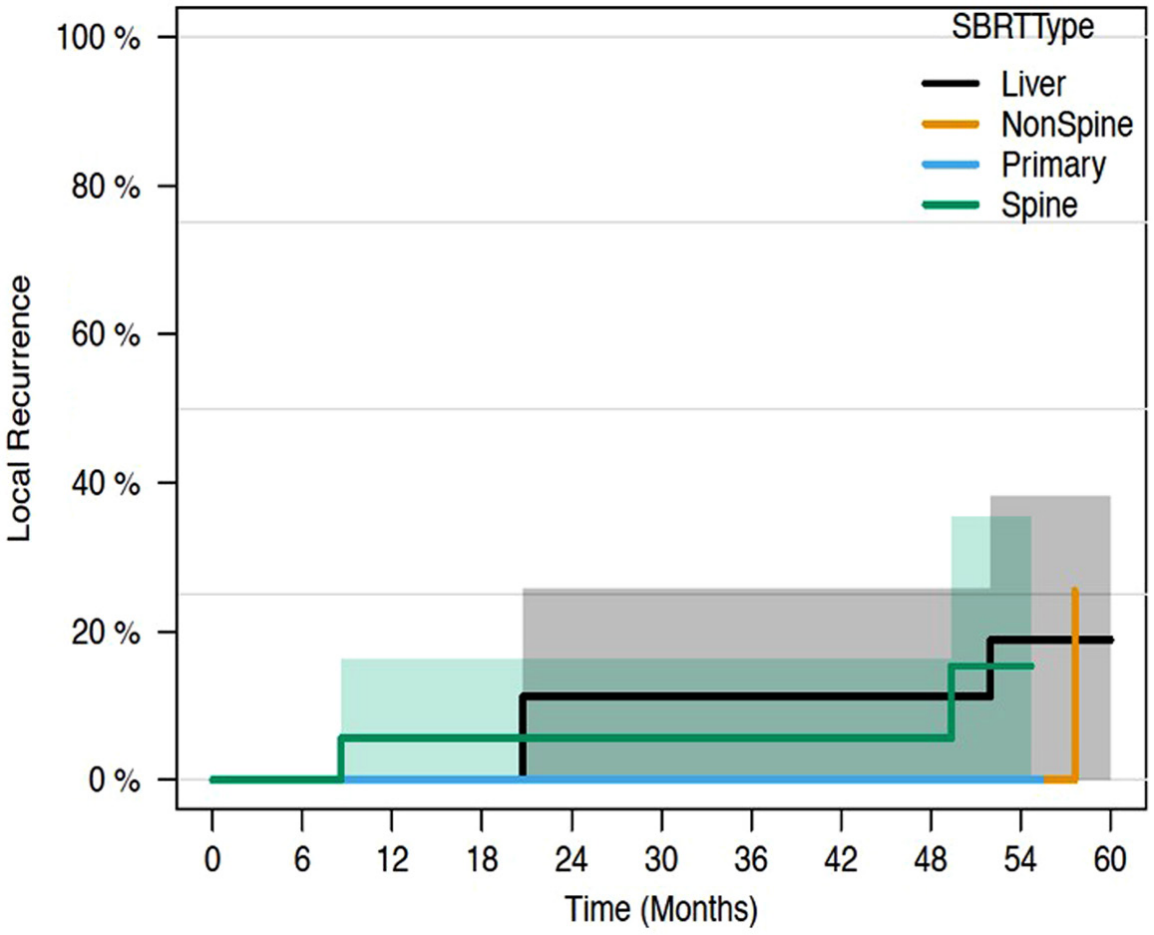
Local recurrence rates stratified by SBRT site.

Figure 4 describes local failure based on anatomic locations treated with SBRT. Rates of local recurrence at 1 and 2 years for liver metastases were 0% and 11% (7.4–25.8%); for non-spine bone metastases, they were 0% and 0%; for spine metastases, they were 5.6% (5.4–16.3%) and 5.6% (5.4–16.3%); and for the primary site, they were 0% and 0%, respectively.

### Toxicity

Acute and late toxicities attributed to either SBRT or PRRT are outlined in Table 3. Radiation toxicity is associated with the target organ and surrounding organs at risk. Specific to spine SBRT, no patient developed a pain flare or radiation myelopathy. No liver toxicity, either radiographic or biochemical, was attributable to SBRT. One case of chest-wall fibrosis/pain was identified, related to the radiation treatment volume. One patient with extensive vertebral body metastases post-PRRT failure, treated with spine SBRT to multiple symptomatic sites, developed grade 3 thrombocytopenia, which did not resolve.

**Table 3:** Acute and late toxicity attributed to SBRT or PRRT.

	Acute toxicity grade – SBRT			Late toxicity grade – SBRT			Acute toxicity grade – PRRT			Late toxicity grade – PRRT				
	1	2	3	1	2	3	1	2	3	1	2	3	4	5
Nausea	3	0	0	0	0	0	0	0	0	0	0	0	0	0
Fatigue	2	0	0	0	0	0	1	0	0	0	0	0	0	0
Pain	0	0	0	0	2	0	1	0	0	0	0	0	0	0
Anemia	1	0	0	1	1	0	5	1	0	5	3	0	0	0
Thrombocytopenia	0	0	1	1	0	1	5	3	1	6	2	1	0	0
Lymphopenia	1	0	0	1	0	0	5	0	0	2	0	0	0	0
Elevated AST	0	0	0	0	0	0	1	0	0	4	1	0	0	0
Elevated ALT	0	0	0	0	0	0	1	0	0	4	1	0	0	0
Elevated ALP	0	0	0	0	0	0	3	0	0	4	2	2	0	0
Elevated bilirubin	0	0	0	0	0	0	0	0	0	0	2	0	2	0
Leukemia	0	0	0	0	0	0	0	0	0	0	0	0	0	1
Myelopathy	0	0	0	0	0	0	0	0	0	0	0	0	0	0

AST, aspartate aminotransferase; ALT, alanine transaminase; ALP, alkaline phosphatase; PRRT, peptide receptor radionuclide therapy; SBRT, stereotactic body radiation therapy.

No unexpected significant acute toxicities were attributable to PRRT. Grade 4 elevated bilirubin levels were identified in two patients as late toxicity. This may be attributable to PRRT +/− SBRT; however, these patients also both had multiple courses of trans-arterial embolization and had notable intra-hepatic progression. One patient developed leukemia post PRRT; SBRT was delivered for local control to progressive neuroendocrine liver metastases after the diagnosis of leukemia was established.

## Discussion

Current treatment modalities for NEN are intended to maximize tumor control while maintaining patients’ quality of life by minimizing side effects. Even in the presence of metastatic disease, patients with well-differentiated grade 1 or 2 NEN can anticipate prolonged survival, prompting the need for multidisciplinary care. Surgical resection is considered for cytoreduction as well as local control at primary sites of disease (Chan *et al.* 2018). Local management of unresectable liver metastases, such as transarterial (radio)embolization (TA(R)E) and radiofrequency ablation (RFA), can be considered. SBRT is an additional option for treating progressive metastases not only from the liver but across all body sites, with low rates of acute and late toxicities (Chen *et al.* 2021, Hudson *et al.* 2022, Oliver *et al.* 2023). In the setting of diffuse disease progression following SSA treatment for grade 1 and 2 disease, PRRT is indicated (Strosberg *et al.* 2017). Despite the excellent results with the use of PRRT as demonstrated in NETTER-1, the heterogeneous nature of neuroendocrine tumors provides further opportunities to optimize the use of this modality and potentially expand the scope of patients who may benefit. Combined SSTR and FDG PET imaging may be an effective biomarker to predict PRRT outcomes. The NETPET prognostic grading scheme utilized both SSTR and FDG imaging to subclassify patients into appropriate categories for treatment with PRRT (Chan *et al.* 2017). In patients with grade 3 NEN, it was demonstrated that baseline FDG PET was predictive of both PFS and OS (Zhang *et al.* 2019). Combined SSTR/FDG imaging may not only provide a rationale for augmenting PRRT in patients but also provide the specific targets that may need escalated therapy. SBRT boost to SSTR- or FDG-avid disease may extend the option of PRRT to more patients or contribute to improved control rates.

The dose–effect relationship of SBRT is well-established in many solid tumors, including liver metastases (Ohri *et al.* 2021). There appears to be an absorbed dose threshold for response using PRRT for pancreatic neuroendocrine tumors (Ilan *et al.* 2015), and advanced image quantification in nuclear medicine is driving progress toward personalized PRRT dosimetry (Lassmann & Eberlein, 2018). One strategy for consideration may be the utilization of a stereotactic external beam radiation boost to limited sites of underdosing post PRRT. This strategy may provide the needed local control at sites of underdosing without the need to escalate the systemic dose of PRRT delivered to achieve a similar result. Accurate PRRT dosimetry involves imaging at intervals to track residual radioactivity and calculate the total integrated dose, but it can be simplified by estimating the integrated activity from imaging data acquired at a single time point (Hou *et al.* 2021). Simplified dosimetry methods make it more feasible to integrate PRRT and SBRT into complex multimodality regimens. Furthermore, in addition to maximizing the radiation dose to specific target volumes, other benefits may be realized specific to NEN patients, as external beam radiation can upregulate the expression of SSTR2 and the uptake of radiolabeled SSA in neuroendocrine and small cell lung cancer cells (Oddstig *et al.* 2006), which may influence the appropriate timing of therapy.

Even in the absence of potential biomarkers or treatment synergy, we can predict patients who may benefit from a combined therapeutic approach. Prior research has demonstrated that PRRT is effective at controlling bone metastases of gastroenteropancreatic neuroendocrine tumors and is associated with a long OS and reduction in pain (Sabet *et al.* 2013). However, there are subtleties in the management of spine metastases that need to be addressed, given the potential for catastrophic patient outcomes should progressive disease result in a spinal cord compression. The SC.24 clinical trial established spine SBRT as the standard of care for patients with painful vertebral body metastases (Sahgal *et al.* 2021). Given the inherent risk of high-dose radiation to the critical neural structures such as the spinal cord or thecal sac, sharp dose gradients are required to optimize the dose to the tumor while respecting the constraints of associated organs at risk. Epidural disease is a known risk factor for local failure (Zeng *et al.* 2023). Aggressive epidural resection in combination with SBRT can further improve local control, highlighting the importance of dosimetry data to achieve dose gradients required for adequate local control (Al-Omair *et al.* 2013). To date, submillimeter dosimetry, as seen in external beam radiation (EBRT) planning, is not feasible post-PRRT, making it challenging to know the true dose deposition in the epidural space. In this current study, no myelopathy was identified in patients treated with PRRT and spine-SBRT, demonstrating safety. Further, in this patient cohort, no patients treated with spine SBRT had a local failure at 2 years post treatment.

Combined radioisotope therapy and EBRT in other tumor types have been evaluated. Most data is retrospective in nature, with few ongoing prospective trials currently in enrollment. A feasibility study showed that [177Lu]Lu-DOTA0-Tyr3-octreotate followed by EBRT was well tolerated in patients with unresectable meningioma (Kreissl *et al.* 2012, Hartrampf *et al.* 2020). A prospective trial compared the analgesic effectiveness of samarium-153 alone or combined with EBRT in prostate cancer patients with bone metastases (Baczyk *et al.* 2013). Radium-223 combined with SBRT or other EBRT showed improved OS in patients with osteoblastic bone metastases (Anderson *et al.* 2020). The RAVENS trial is currently underway, comparing SBRT alone or SBRT with radium-223 in prostate cancer patients with bone metastases (Hasan *et al.* 2020). More applicable to patients with neuroendocrine tumors, a small series evaluating the sequential administration of TARE and liver EBRT for patients with locally advanced HCC demonstrates the tolerability of therapy and is suggestive of improved local control and survival (Hardy-Abeloos *et al.* 2019, Liu *et al.* 2021). With any combination therapy, there needs to be an understanding of the dose–effect relationship of both modalities in malignant and normal tissues.

This is the first published study describing patients treated with both PRRT and SBRT for neuroendocrine tumors. The modest OS in this cohort of 19.6 months is likely reflective both of the clinical nature of the patients treated and the measurement of OS calculated from the last treatment delivered to avoid immortal time bias. There may be inherent clinical behavior differences in the disease treated initially with SBRT then PRRT (typically initial oligometastatic/oligoprogressive disease followed by systemic progression) as opposed to those treated with PRRT then SBRT (systemic progression with subsequent oligoprogression) that influence both local control rates as well as PFS and OS. Excellent local control of disease treated with SBRT was observed, which may be relevant when looking at the factors that may influence oligoprogressive disease in patients treated with PRRT. The local control benefit is most evident in the patients treated initially with PRRT and subsequent SBRT. The number treated in this group is small; there may be unrecognized confounding factors. However, the benefit of this treatment order is hypothesis generating and may influence the timing of treatments in subsequent trial design. Despite the limitation of this evaluation that SBRT and PRRT were not co-administered, we highlight the favorable tolerability and low incidence of both acute and long-term adverse effects in line with prior published toxicity data for both PRRT and SBRT as an individual treatment. This is comparable to current evidence of safety of PRRT (Strosberg *et al.* 2017) or SBRT (Hudson *et al.* 2022) as sole therapy and supports further research into this potential combined therapy.

## Declaration of interest

JR: none to declare; SN: none to declare; HC: none to declare; SS: research support from Novartis Oncology and Ipsen. Honorarium from Novartis Oncology and Ipsen; JH: honorarium from Novartis Oncology and Ipsen; CL: honorarium from Novartis Oncology, Ipsen, Amgen, Gilead, Taiho; SM: research Support from Novartis Oncology and Ipsen. Honorarium from Novartis Oncology and Ipsen.

## Funding

This work did not receive any specific grant from any funding agency in the public, commercial, or not-for-profit sector.
